# Impact of Persistent Cytomegalovirus Infection on Dynamic Changes in Human Immune System Profile

**DOI:** 10.1371/journal.pone.0151965

**Published:** 2016-03-18

**Authors:** Rosanna Vescovini, Anna Rita Telera, Mario Pedrazzoni, Barbara Abbate, Pietro Rossetti, Ignazio Verzicco, Maria Cristina Arcangeletti, Maria Cristina Medici, Adriana Calderaro, Riccardo Volpi, Paolo Sansoni, Francesco Fausto Fagnoni

**Affiliations:** 1 Department of Clinical and Experimental Medicine, University of Parma, via Gramsci 14, 43126, Parma, Italy; 2 Immunohematology and Transfusion Center, Hospital of Parma, via Gramsci 14, 43126, Parma, Italy; University of St Andrews, UNITED KINGDOM

## Abstract

Human cytomegalovirus (HCMV) imprints the immune system after primary infection, however its effect during chronic infection still needs to be deciphered. In this study we report the variation of blood cell count along with anti-HCMV IgG and T cell responses to pp-65 and IE-1 antigens, that occurred after an interval of five years in a cohort of 25 seropositive healthy adults. We found increased anti-viral IgG antibody responses and intracellular interferon-gamma secreting CD8+ T cell responses to pp-65: a result consistent with memory inflation. With the only exception of shortage in naive CD8+ T cells most memory T cell subsets as well as total CD8+ T cells, T cells, lymphocytes, monocytes and leukocytes had increased. By contrast, none of the cell types tested were found to have increased in 14 subjects stably seronegative. Rather, in addition to a shortage in naive CD8+ T cells, also memory T cell subsets and most other cell types decreased, either in a statistically significant or non-significant manner. The trend of T cell pool representation with regard to CD4/CD8 ratio was in the opposing directions depending on HCMV serology. Globally, this study demonstrates different dynamic changes of most blood cell types depending on presence or absence of HCMV infection. Therefore, HCMV plays a continual role in modulating homeostasis of blood T cells and a broader expanding effect on other cell populations of lymphoid and myeloid origin.

## Introduction

Human Cytomegalovirus (HCMV) is a common virus infecting a large proportion of the human population with an estimated seroprevalence of 45–90% worldwide [1–3]. Interest in HCMV has concentrated on congenital infection and pathologic conditions characterized by risks of uncontrolled infection. But in normal conditions, the vast majority of people establish a benign infection and viral-immune system interaction may be considered part of the human immune system physiology [4]. Indeed emerging data show that this pathogen has a broad influence on the overall immune profile of healthy individuals [5]. Therefore, how the dynamics of the interactions between HCMV and the host imprints our immune system, and perhaps our physiology more generally, is gaining increasing interest.

One of the most prominent effects of HCMV infection is modulation of the absolute numbers of circulating blood T cell subsets. The profile of the peripheral T cell pool is characterized by expansion of memory T cells after recovery from primary infection, [6,7] and significant differences persist between seronegative and seropositive subjects [8]. Moreover, cross-sectional studies are suggesting that HCMV along with aging induce further increase in memory T cell numbers [9–12]. However, cross-sectional studies may suffer from bias deriving from different effects of the primary HCMV infection at different ages. Therefore, the hypothesis of a distinct dynamics between subjects carrying HCMV as compared to others still awaits confirmation through longitudinal studies.

Another prominent aspect of HCMV is the large proportion of adaptive immune resources engaged for its immunosurveillance. In particular, anti-HCMV specific CD4+ and CD8+ T cell responses are broadly targeted and dominate the memory compartment of seropositive subjects [13]. Furthermore, cross-sectional studies limited to a few selected antigens are suggesting that anti-HCMV immune responses may expand with aging [14], thus making seropositive elderly people candidates for a massive load of anti-HCMV immune responses [15]. Longitudinal studies conducted so far have not yet confirmed a real trend towards expansion of memory responses to HCMV. They have all reported a great time-dependent variability, although covering relatively short intervals when compared to the human lifespan [16–19]. Interestingly, studies in the murine model have shown a continuous accumulation of antiviral CD8+ T cells over time, the so-called "memory inflation" phenomenon [20, 21]. These discrepancies need further extended longitudinal studies.

In this study, we approached the dynamics of anti-viral immune responses along with T cell subsets distribution and white cells count, by observing a cohort of subjects stably carrying HCMV, before and after an interval of approximately five years. Some antigen-specific responses and even some white cell parameter had increased. Whereas a cohort of CMV seronegative donors, acting as a control for the seropositive group, revealed a relative decline in white cell numbers.

## Materials and Methods

### Subjects and blood samples

**Fig 1:**
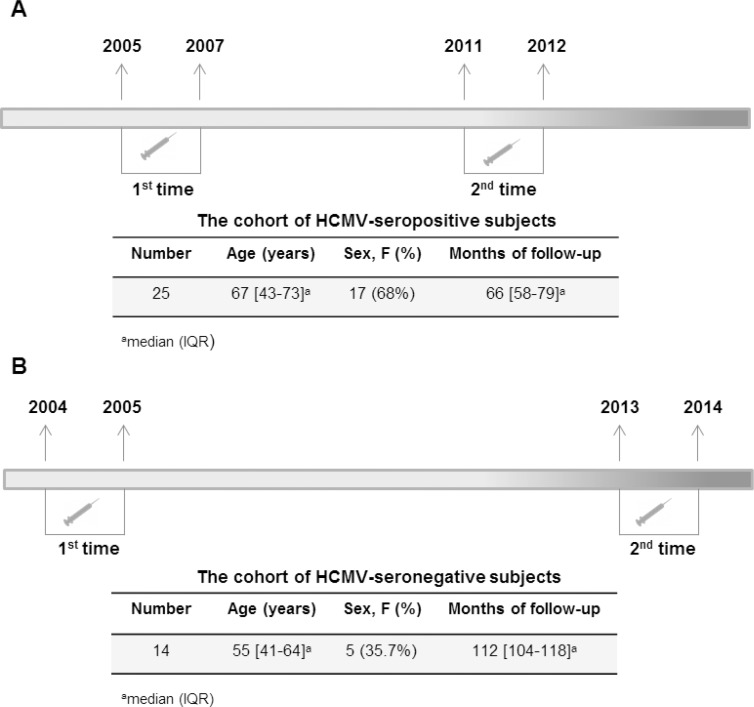
Description of the study. Follow-up times and characteristics of the HCMV-seropositive subjects (A) and of the HCMV-seronegative subjects (B).

The study was performed after approval from the Ethics Committee of the University of Parma. HCMV-seropositive subjects, living in Parma were studied for the first time in the period 2005–2007. Subsequently, they were evaluated again between 2011 and 2012. Likewise, HCMV-seronegative subjects living in Parma and evaluated for the first time in the period 2004–2005, were evaluated again between 2012 and 2013. At both time points, all subjects were required to answer standardized questionnaires to assess clinical history, current disease and current medication list; they were subjected to physical examination at the same time. Exclusion criteria were: evidence of endocrine, autoimmune and neoplastic diseases, acute infections or very recent infections (in the last 2 months), renal and liver failure, use of medications known to modulate the immune responses (steroids, non-steroidal anti-inflammatory agents, acetyl salicylic acid >100mg/day or other immunosuppressive drugs), current smoking habit. Among the subjects contacted the first time, 25 HCMV-seropositive and 14 HCMV-seronegative subjects confirmed their willingness to participate in the study at the second time. After written, informed consent, all these donors had blood drawn to perform the analyses (Fig 1). The experimental design was to draw and test each donor individually and independently from each other, except for Antibody detection, as specified below.

### CMV-IgG and IgM detection

To determine anti-HCMV IgM and IgG Ab levels plasma samples drawn from all donors at both time points were frozen and stored. To compare HCMV-IgG levels across the two time points, to avoid inter-assay variability, frozen plasma samples collected from all HCMV-seropositive subjects at both times of the study were thawed and tested in a single session at the University Hospital of Parma (Virological Unit). Indirect chemiluminescence immunoassays, CLIA, were used for the quantitative evaluation of anti-CMV IgM and IgG (Liaison HCMV IgM and HCMV IgG assays, DiaSorin, Vercelli, Italy). Samples were analyzed by a photomultiplier Liaison (DiaSorin), following the manufacturer’s protocol. The serostatus of HCMV-seronegative subjects was retested the second time to exclude subjects who had undergone seroconversion.

### Blood count and Immunophenotyping

#### Whole blood cell counts

All analyses were carried out in our quality certified hematology laboratory: the automated analyser was a MAXM (Instrumentation Laboratory, Bedford, Massachusetts, USA) at the first collection time for all subjects, and it was a XT-2000i (Sysmex Corporation, Kobe, Japan) at the second time point for all subjects (Fig 1).

#### Flow cytometry analysis

Surface staining was performed on freshly drawn heparinized whole blood using the following mAbs: anti-CD28-FITC, anti-CD95-PE, anti-CD3-PerCP and anti-CD8-APC or anti-CD4-APC (all from BD Biosciences, San Jose, CA). One hundred microliters of blood were incubated with saturating amounts of the mAbs for 20 minutes on ice and then were lysed with FACS Lysing Solution (BD Biosciences). Six-parameter FCM acquisition and analysis were performed on a two-laser FACSCalibur instrument (BD Biosciences) using CellQuest software (BD Biosciences). Files were first gated on lymphocytes, identified by characteristic forward angle and side scatter profiles and CD3+CD4+ T cell or CD3+CD8+ T cell subsets were gated into naïve (CD28+CD95-), central memory (CM, CD28+CD95hi), and effector/effector memory (EM, CD28-CD95hi) subsets (S1 Fig), as previously published [22]. Isotype-matched irrelevant antibodies were used to set fluorescence markers and to identify non-specific binding. Standardisation of analysis and comparability of results were related to the use of reagents from the same producing company, instrument compensation and gating strategy performed by the same operator at both time points for all subjects. Absolute counts of T cell subsets were obtained by dual platform based on total lymphocyte counts from blood cell counts and frequencies of CD4+ and CD8+ T cell quantified by flow cytometry. Thus, the absolute numbers of total, naïve, CM and EM CD4+ and CD8+ T cells per μL of peripheral blood were calculated multiplying lymphocyte counts by the frequency of different subsets in the lymphocyte gate.

### Assessment of HCMV-specific T cell responses by intracellular cytokine staining (ICS)

In all HCMV-seropositive subjects, at both times of the study, anti-HCMV T cell responses were assessed individually, as the blood as drawn, as previously described [15]. Peripheral blood mononuclear cells (PBMC_s_) were obtained by Ficoll density gradient centrifugation (Biocoll Separating Solution; Biochrom AG, Berlin) from freshly drawn venous blood. After washing with phosphate-buffered saline (PBS), PBMC_s_ were resuspended in RPMI 1640 medium supplemented with 10% fetal calf serum (FCS), 2 mmol/L L-glutamine, 100μg/mL streptomycin and 100 units/mL penicillin (complete medium). In all experiments the cells were immediately stimulated with PepMix pp65 and PepMix IE-1 (JPT Peptide Technologies, Berlin, Germany), spanning the 65kDa lower matrix phosphoprotein and the 55kDa immediate-early protein 1 respectively, and consisting of 15 amino acid long peptides, overlapped by 11 amino acids. Stimulation and intracellular cytokine detection was performed in accordance with the protocol recommended by JPT Peptide Technologies and well described. Briefly, 10^6^ PBMC_s_ were placed in 15-ml conical polypropylene tubes (Corning Incorporated, NY) in a final volume of 500 μL of complete medium and incubated with one test volume of each PepMix and 1 μg each of the costimulatory mAbs CD28 and CD49d (BD Biosciences). Negative controls (incubation with anti-CD28/CD49d but not with PepMixes) were included in every experiment to detect spontaneous production of cytokines. After 1h of incubation in a standard incubator (37°C, humidified CO_2_ atmosphere) each tube received 500 μL of complete medium containing the protein transport inhibitor monensin (BD Golgi Stop, BD Biosciences). After an additional 4 h, PBMC_s_ were fixed, permeabilized and then stained with saturating amounts of antibodies anti-IFN-γ, anti-CD4 and anti-CD8 (all from BD Biosciences). The samples were acquired at the FACSCalibur cytometer, and analyzed as described above. Files were gated on lymphocytes and cytokine–secreting populations were defined as the percentage of the IFN-γ^+^ events gated on CD4+ or CD8+ T cell population minus the percentage of the events falling into the same region in the corresponding control sample. Between 0.15 and 0.4 x 10^6^ total events were acquired and the percentage threshold of positivity was set for each stimulated sample at 0.03%, after subtraction of the value for unstimulated sample The absolute numbers of CMV-specific CD4+ or CD8+ T cells per μL of peripheral blood were calculated multiplying lymphocyte counts by the frequency of CD4+ or CD8+ T cells in the lymphocyte gate and by the frequency of IFN-γ producing cells within total CD4+ or CD8+. Standardisation of analysis and comparability of results were still dependent on the use of reagents from the same producing company, instrument compensation and gating strategy performed by the same operator at both time points for all subjects.

### Statistical analysis

For longitudinal time points, the Wilcoxon signed-rank nonparametric was used to evaluate changes within antigen-specific responses and all cell types. *p* less than 0.05 was considered significant in all instances.

## Results

### Follow-up of anti-HCMV specific responses

**Fig 2:**
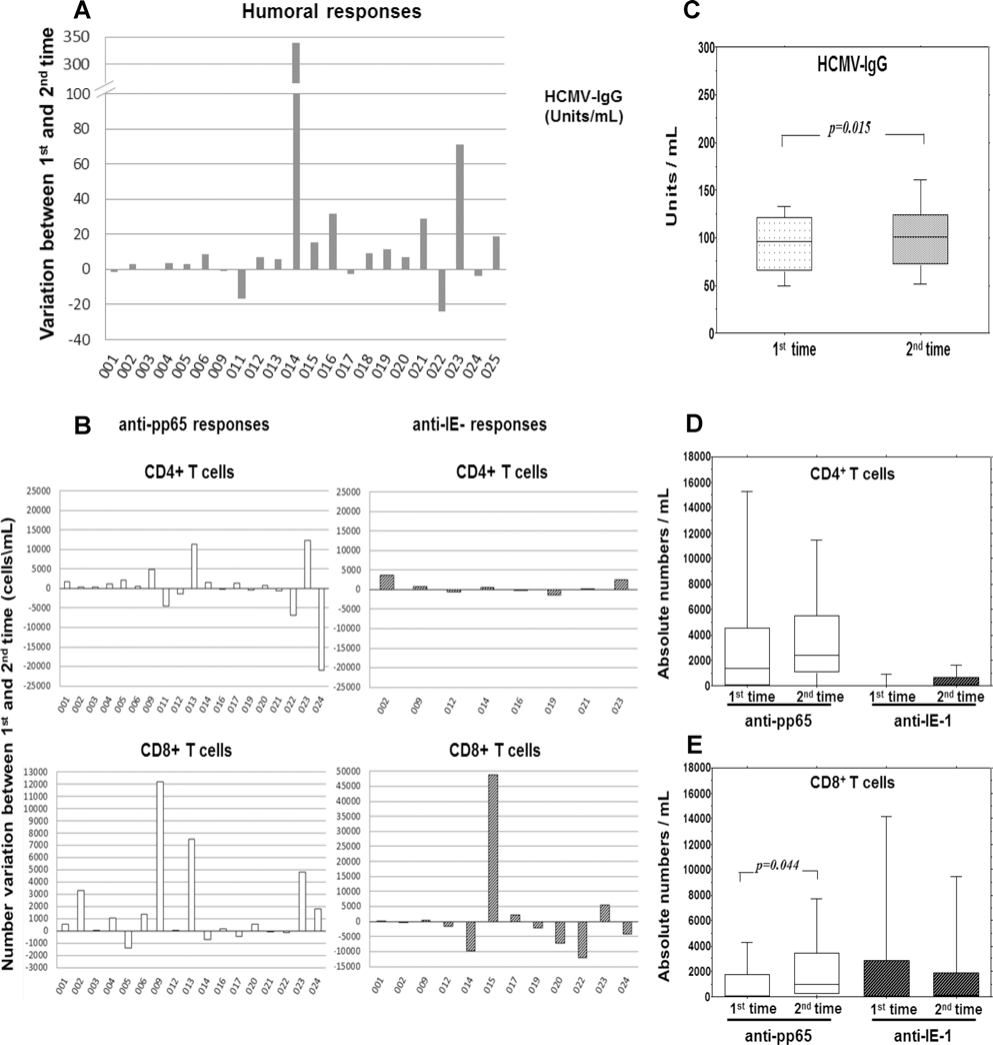
Follow-up of HCMV-specific responses. (A) Unit variation of the HCMV-specific IgG Ab between the first and the second time point of the follow-up. (B) Number variations of anti-pp65 and anti-IE-1 CD4 and CD8 T cell responses, evaluated as differences in intracellular IFN-γ positive T cells after stimulation of freshly isolated PBMCs with HCMV synthetic peptides, between the first and the second time point of the follow-up. (C) Levels of HCMV-specific IgG Ab at the two time points of the follow-up. (D) Absolute numbers of HCMV-specific CD4 and CD8 T cell responses at the two time points of the follow-up. In all the panels the individual variations are shown in individual subjects sorted with ascending order of age. In panels C and D, the box plots show the median, 25^th^ and 75^th^ percentiles, and range. The Wilcoxon signed-rank nonparametric tests were used to derive *p* values.

We performed the follow-up of anti-HCMV specific responses with different approaches for the two types of response. For humoral responses, all antibody data were generated at the same time using frozen plasma samples (because it is common knowledge that cryopreservation does not alter antibody content). By contrast, for cellular data, we chose to test individually fresh samples, in order to: (i) obtain ex-vivo data as close as possible to in vivo function; (ii) avoid altering conditions such as freezing, thawing, and long-term storage, all of which may affect viability, recovery and cell function. We measured IgM and IgG responses to HCMV in the group of subjects described in Fig 1A. All donors were negative for the anti-HCMV IgM at both time points of the study and this result did not support undergoing reactivation events or inter-current infections. Considering the variations of anti-HCMV IgG levels we found an increase in 15 out of 22 tested subjects (for the subjects 007, 008 and 010 the determinations were only qualitative), a decrease in 6 out of 22 and only one subject with a null variation (Fig 2A).

Likewise, the variations of the anti-pp65 and IE-1 intracellular interferon-gamma responses detected in this group of subjects, were broadly heterogeneous, both in tendency and intensity (Fig 2B). Our data confirmed the well-known immunodominance of the pp65 viral protein, with 19 out of 21 tested subjects with a CD4+ anti-pp65 response (8/21 with a CD4+ anti-IE1 response) and 17 out of 21 subjects with a CD8+ anti-pp65 response (12/21 with a CD8+ anti-IE1 response). Interestingly, among the CD8+ anti-pp65 responders, in 12 out of 17 subjects the CD8+anti-pp65 responses increased between the first and the second time points of the study, while in 5 out of 17 subjects the responses decreased.

Collectively, after a period of about 5 years, in spite of a common apparently erratic variability, non-parametric paired comparison of the two time points showed a significant trend to increase (p<0.05) in anti-HCMV IgG and CD8+ anti-pp65 responses (Fig 2C and 2E). These results showed that in adulthood, after an interval of five years, the prevailing trend of some anti-HCMV adaptive immune responses such as humoral IgG and CD8 pp65 responses was toward a slight but significant expansion.

### Follow-up of T cells and white blood cells numbers in peripheral blood of subjects seropositive for HCMV

**Fig 3:**
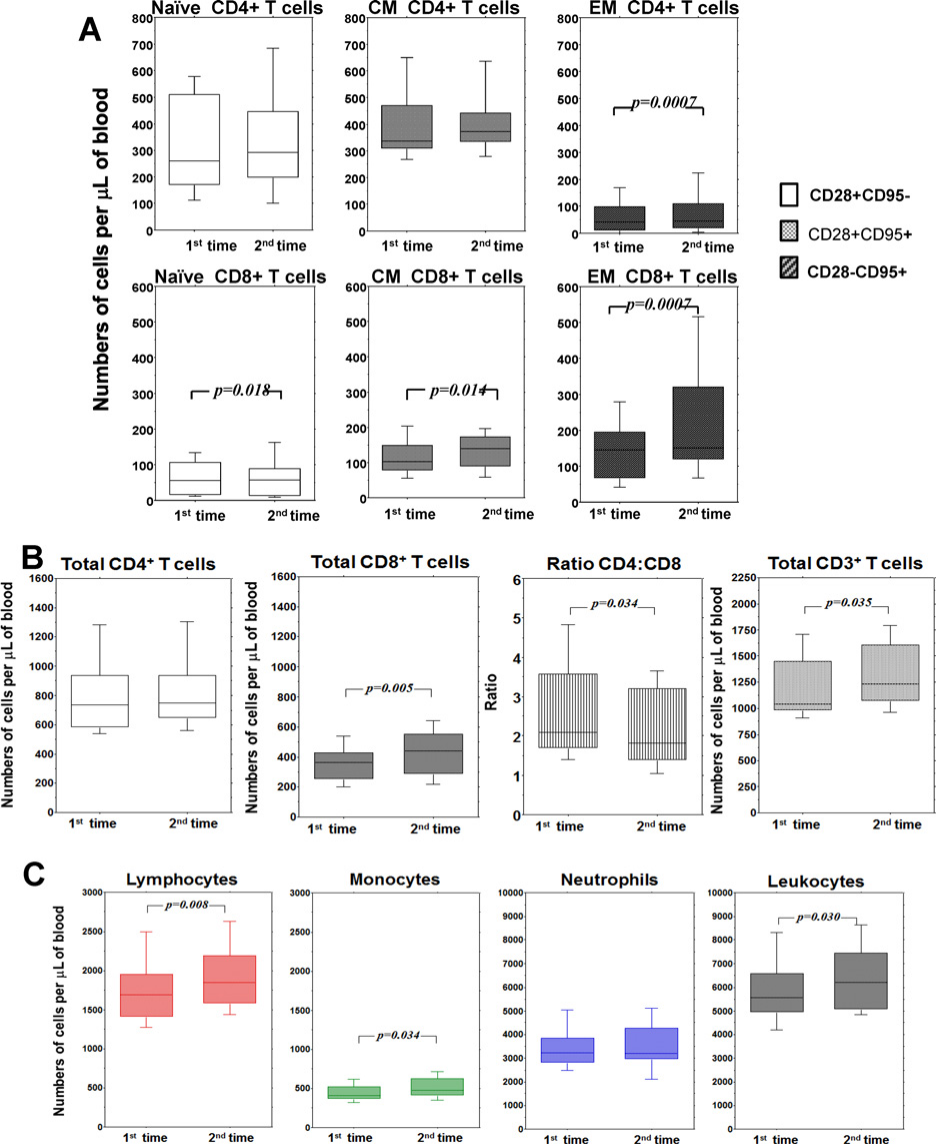
Follow-up of T cells and white blood cells numbers in peripheral blood of HCMV-seropositive subjects. (A) Absolute numbers of naïve, central memory (CM) and effector memory (EM), among CD4+ and CD8+ T cells at the two time points of the follow-up. (B) Absolute numbers of total CD4+, total CD8+, ratio CD4:CD8 and total CD3+ at the two time points of the follow-up. (C) Absolute numbers of leukocytes, monocytes, neutrophils and leukocytes at the two time points of the follow-up. In all panels, the box plots show the median, 25^th^ and 75^th^ percentiles, and range. The Wilcoxon signed-rank nonparametric tests were used to derive *p* values.

The variation observed in each HCMV-seropositive subject was heterogeneous also in the case of absolute numbers of CD4+ and CD8+ T cell subsets (S2 Fig). In particular, variation of naive and central memory CD4+ T cells was not only heterogeneous but also erratic, without a statistically significant tendency (Fig 3A).

By contrast, in the case of CD4+CD28- T cells there was a clear prevailing tendency to increase (Fig 3A). Similarly, the dynamics of naive (decrease) and memory (increase) CD8+ T cells were clearly significant and divergent (Fig 3A). But the quantitative effect of the decrease of naive CD8+ T cells was marginal, whereas the increase in memory CD8+ T cells was substantial (Fig 3A). Thus, accumulation of memory T cells exceeded the loss of naive CD8 T cells and the overall effect was a significant increase in total CD8+ T cells, and even in total CD3+ T cells (Fig 3B). Interestingly, inflation of CD8+ T cells driven by memory subsets was not accompanied by significant adjustment of CD4+ T cells (the increase of CD4+CD28- T cells was not sufficient to increase total CD4+ T cells), therefore the CD4/CD8 ratio was also significantly reduced (Fig 3B). Finally, when we took a look at whole blood cell counts (S3 Fig), we were surprised to see an increase even in total lymphocytes, monocytes and total white blood cells (Fig 3C). The trend of total lymphocytes was consistent with that of total T cells, which are the main lymphocyte population, but the increase in monocytes was an effect obviously independent from T cells and lymphocytes. In the end, the overall view of dynamics of peripheral blood cell populations evaluated in our study indicates that, in spite of great individual variability and apart from the decrease in naive CD8+ T cells, the prevailing long-term tendency of memory T cells, CD8+ T cells, T cells, lymphocytes, monocytes and white blood cells in HCMV seropositive subjects was to increase.

### Follow-up of T cells and white blood cells numbers in peripheral blood of subjects seronegative for HCMV

**Fig 4:**
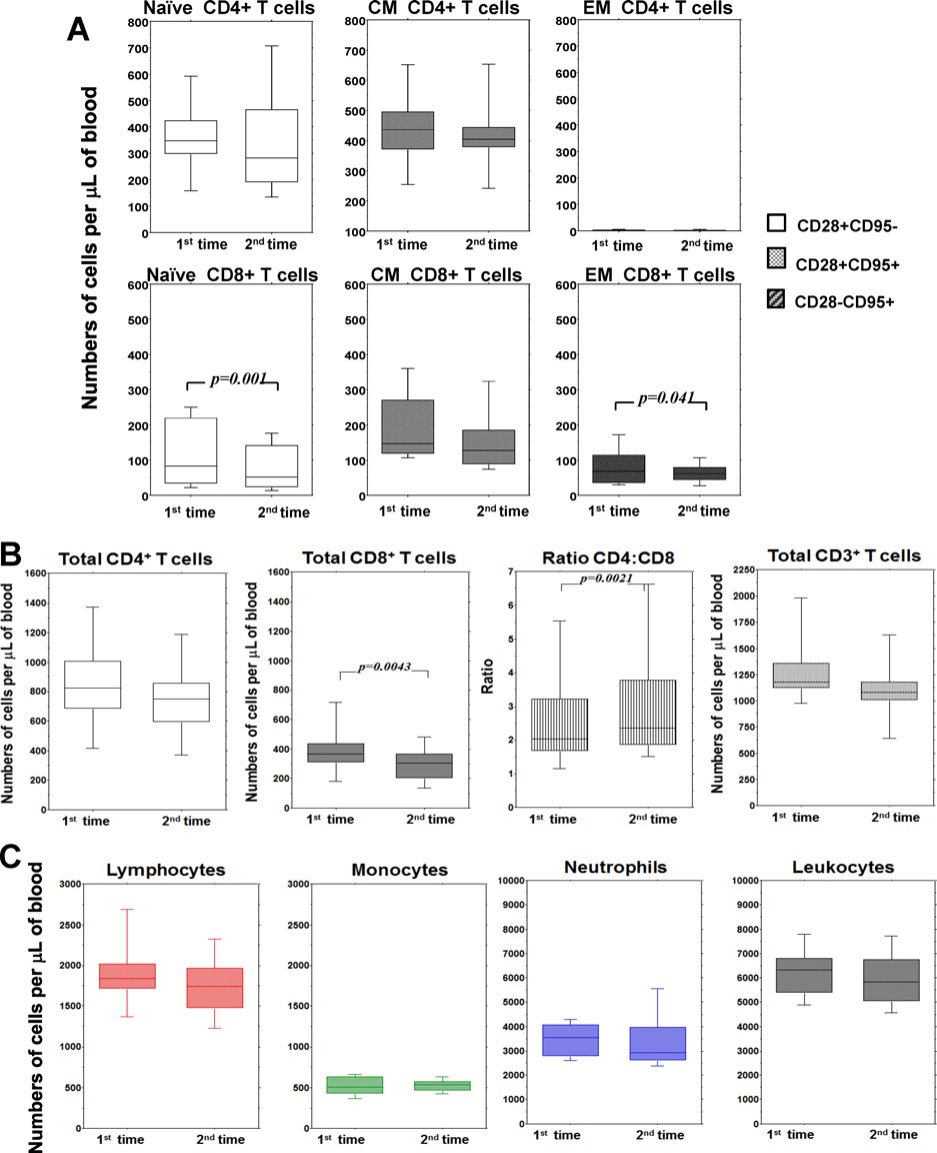
Follow-up of T cells and white blood cells numbers in peripheral blood of HCMV-seronegative subjects. (A) **A**bsolute numbers of naïve, central memory (CM) and effector memory (EM), among CD4+ and CD8+ T cells at the two time points of the follow-up. (B) Absolute numbers of total CD4+, total CD8+, ratio CD4:CD8 and total CD3+ at the two time points of the follow-up. (C) Absolute numbers of leukocytes, monocytes, neutrophils and leukocytes at the two time points of the follow-up. In all panels, the box plots show the median, 25^th^ and 75^th^ percentiles, and range. The Wilcoxon signed-rank nonparametric tests were used to derive *p* values.

An important feature of this study was the inclusion of a cohort of CMV seronegative donors, which acts as a control for the seropositive group. We examined fourteen seronegative subjects on average ten years younger than seropositive, at two time points separated by an even more extended interval (almost ten years, Fig 1B). Broad individual variability was still a common feature (S4 and S5 Figs). The other feature shared with the HCMV seropositive group was a homogeneous and consistent decrease in CD8+, but not CD4+, naive T cells (Fig 4A).

By contrast, the behavior of memory T cells was clearly different. The tendency of central memory CD4+ and CD8+ T cells was an apparent, even if not significant, decrease. Effector memory cells (CD28-) remained absent within the CD4+ subset and significantly decreased within the CD8+ subset (Fig 4A). This latter effect also induced quantitative reduction of total CD8+ T cell counts (Fig 4B) and increase of the CD4/CD8 ratio, even if reduction in total T cell number was not statistically significant. Finally, the counts of lymphocytes and white blood cells showed a slight trend to diminution, but the final effect with this limited number of subjects did not reach statistical significance (Fig 4C). Therefore, in the absence of HCMV infection, the entire CD8+ T cells (not only naive but also memory) decreased with time. Also, other cell types had not increased, but rather decreased, even if not in a statistically significant manner.

We did not directly compare by statistical analysis seropositive vs. seronegative groups because of their different interval between the two points of observation. Nevertheless, in many instances there was clear evidence of a distinctive behavior in relation to anti-HCMV serological status (Figs 3 and 4). The long-term tendency to inflate or deflate counts of many cell types and to decrease or increase CD4/CD8 ratio, respectively, appeared divergent depending on HCMV serology.

## Discussion

The data reported in this study demonstrate that HCMV chronic infection significantly influences the long-term maturation of the immune system profile. People seropositive for HCMV showed a consistent tendency to inflate several T cell memory subsets, lymphocytes and even monocytes. By contrast, stably seronegative subjects showed opposite trends. Such dichotomy suggests that the long-term increase in T cells and leukocytes in peripheral blood is specific to seropositive subjects.

The inflating dynamic effect involved also a concomitant increase in anti-HCMV antibodies concentration and anti-pp65 CD8+ specific immune responses.

These results are consistent with the emerging notion that the immunological memory to HCMV is peculiar. The common tenet is that memory T cells against hundreds of different ubiquitous pathogens once generated are then stably maintained at constant levels through homeostasis [23]. However, cross-sectional studies have shown that the magnitude of peripheral T cell responses to HCMV are even larger in the elderly [14, 15, 24]. Causes and consequences of this unexpected load of anti-HCMV memory T cells are currently under investigation. The great magnitude *per se* of adaptive responses to HCMV is somehow justified by its dimension and complexity [13], whereas expansion seen in the aged still needs to be explained. It could depend on either amplification of responses during long-term infection or intensified responses following primary infection in advanced age, or both. In this study, even if limited to only two time points and few antigen specificities, even in the presence of a broad individual variability, we found a weak but significant probability to count higher numbers of anti-pp65 CD8+ in blood after five years (Fig 2B and 2D). Although expected on the basis of the "memory inflation" phenomenon reported in the mouse model [20, 21], this finding is novel in humans. Indeed, previously published longitudinal studies, with a shorter interval of observation lasting from months [16, 17] to three years [18, 19], failed to find this same tendency of antigen-specific immune responses to expand during the chronic phase of infection. Previous studies were probably insufficient to detect the "memory inflation" dynamic because of the great individual variability of responses, the wide time-dependent fluctuations of frequency of antigen-specific memory T cells, and shorter interval of observation. Another explanation for our results, alternative to "memory inflation", would be a displacement of T cells to blood, as they represent only 2–2.5% of total T cells in the body [23]. But it should be noted that inflation was not limited to memory CD8+ T cells reactive to pp65 antigen because also the concentration of antibody against HCMV was increased without potential influence by compartment displacement. In both cases of cellular and antibody responses, tendency to accumulate was not univocal but simply prevalent because of the broad heterogeneity and fluctuation of responses already seen in previous studies.

A previous study [25] observed that anti-CMV IgG were stable over a decade, however it should be noted that the median age of donor tested was higher than in our cohort, that median IgG concentration was lower and that in 8 cases out of twelve the IgG levels were also slightly increased.

Therefore, although preliminary and limited, our results taken together constitute consistent proof of the concept that not only CD8+ T cells, as described in mice, but also humoral memory to HCMV tended toward an accumulation in the long-term.

HCMV-dependent increase was even more evident when considering cell dynamics regardless of antigen specificity. This trend involved memory T cells, mostly with the effector phenotype. Moreover the impact of carrying HCMV revealed to be broader than expected. Indeed, not only memory T cell subsets, but also total CD8+ T cells, total T cells, total lymphocytes, and even monocytes and total leukocytes showed a slight but significant probability to increase in five years. Unfortunately, we did not evaluate B cells and NK cells, but their contribution to increase in total lymphocytes can be presumed based on previous reports from cross-sectional studies [11, 26, 27]. On the other hand, the relevance of HCMV on monocyte counts, at least to our knowledge, is unprecedented and opens a new window of interest on the possible effects of HCMV on generation and homeostasis of the monocyte population and possibly on dendritic precursors of monocytic origin. Thus, seropositive subjects showed a consistent trend towards increases in many cell types, an effect totally absent in the HCMV-seronegative subjects, acting as control group in spite of their lower age (about twelve years). Interestingly, cell types such as effector-memory CD8+ T cells and total CD8+ T cells showed a more significant trend to decrease in the absence of HCMV, whereas in other instances median numbers tended to reduce, but without reaching statistical significance. The most striking discrepancy was seen in the change of CD4/CD8 ratio, an immunological parameter broadly used when assessing individual immune competence in clinical studies [28, 29]. The opposing trends between the two groups of subjects included in our study demonstrate that change of the CD4/CD8 ratio over time is heavily influenced by HCMV. Finally, these data ultimately demonstrated that the effects of HCMV extend well beyond those related to primary infection. Rather, this study suggests that HCMV plays a continual role in modulating the homeostasis of blood T cells and a broader expanding effect on other cell population of lymphoid and myeloid origin.

The correct representation of the peripheral T cell pool, lymphocytes and white blood cells is essential for surveillance against pathogens. Reduction in the T cell pool is a common aspect of pathological conditions such as human immunodeficiency virus (HIV) infection [30], cytotoxic chemotherapy [31], strong T cell depletion [32], and thymectomy in early age [33]. In this study we have had preliminary evidence that HCMV, a persistent virus requiring stringent immune surveillance to prevent reactivation, induces a dynamic increase in blood cell counts in the long-term. This finding adds new insights into long-term consequences of infection by HCMV [34] and contributes to understanding the development over time of the normal immune system. In other words, the presence or absence in the human body of this lifelong passenger makes a substantial difference not only because of primary infection remnant effects, but also for long-term maturation of the immune system profile. Many implications might emerge from this observation, but in our opinion the strongest is that many aspects of immune physiology and pathology appear to evolve in a very different way in the two worldwide groups of subjects carrying or not carrying this environmental factor.

## Supporting Information

S1 Fig Flow cytometry gating strategy. Representative flow cytometric analysis of one HCMV-seropositive and one HCMV-seronegative donor, showing gating to identify T cell subset populations (Naïve, CM, and EM). The absolute numbers of naïve, CM and EM CD4+ and CD8+ T cells per mL of peripheral blood were calculated as described in Materials and Methods.
(TIF)

S2 Fig Follow-up of CD4 and CD8 T cell subset numbers in peripheral blood of HCMV-seropositive subjects. (A, C, E) Number variations of naïve, central memory (CM) and effector memory (EM), among CD4+ T cells and (B, D, F) among CD8+ T cells. The variations are shown in the 25 HCMV-seropositive subjects (from 001 to 025) sorted with ascending order of age.
(TIF)

S3 Fig Follow-up of T cells and white blood cells numbers in peripheral blood of HCMV-seropositive subjects. (A, B, C, D) Number variations of total CD4+, total CD8+, ratio CD4:CD8 and total CD3+; (E, F, G, H) number variations of leukocytes, monocytes, neutrophils and leukocytes in the individual 25 HCMV-seropositive subjects sorted with ascending order of age.
(TIF)

S4 Fig Follow-up of CD4 and CD8 T cell subset numbers in peripheral blood of HCMV-seronegative subjects. (A, C, E) number variations of naïve, central memory (CM) and effector memory (EM), among CD4+ T cells and (B, D, F) among CD8+ T cells. The variations are shown in the 14 HCMV-seronegative subjects (from 001 to 014) sorted with ascending order of age.
(TIF)

S5 Fig Follow-up of T cells and white blood cells numbers in peripheral blood of HCMV-seronegative subjects. (A, B, C, D) Number variations of total CD4+, total CD8+, ratio CD4:CD8 and total CD3+; (E, F, G, H) number variations of leukocytes, monocytes, neutrophils and leukocytes in the individual 14 HCMV-seronegative subjects sorted with ascending order of age.
(TIF)
