# Supplementary figures and images for: Impact of Persistent Cytomegalovirus Infection on Dynamic Changes in Human Immune System Profile
Source: PLoS One. 2016 Mar 18;11(3):e0151965. doi: 10.1371/journal.pone.0151965 (PMC4798275; doi:10.1371/journal.pone.0151965)

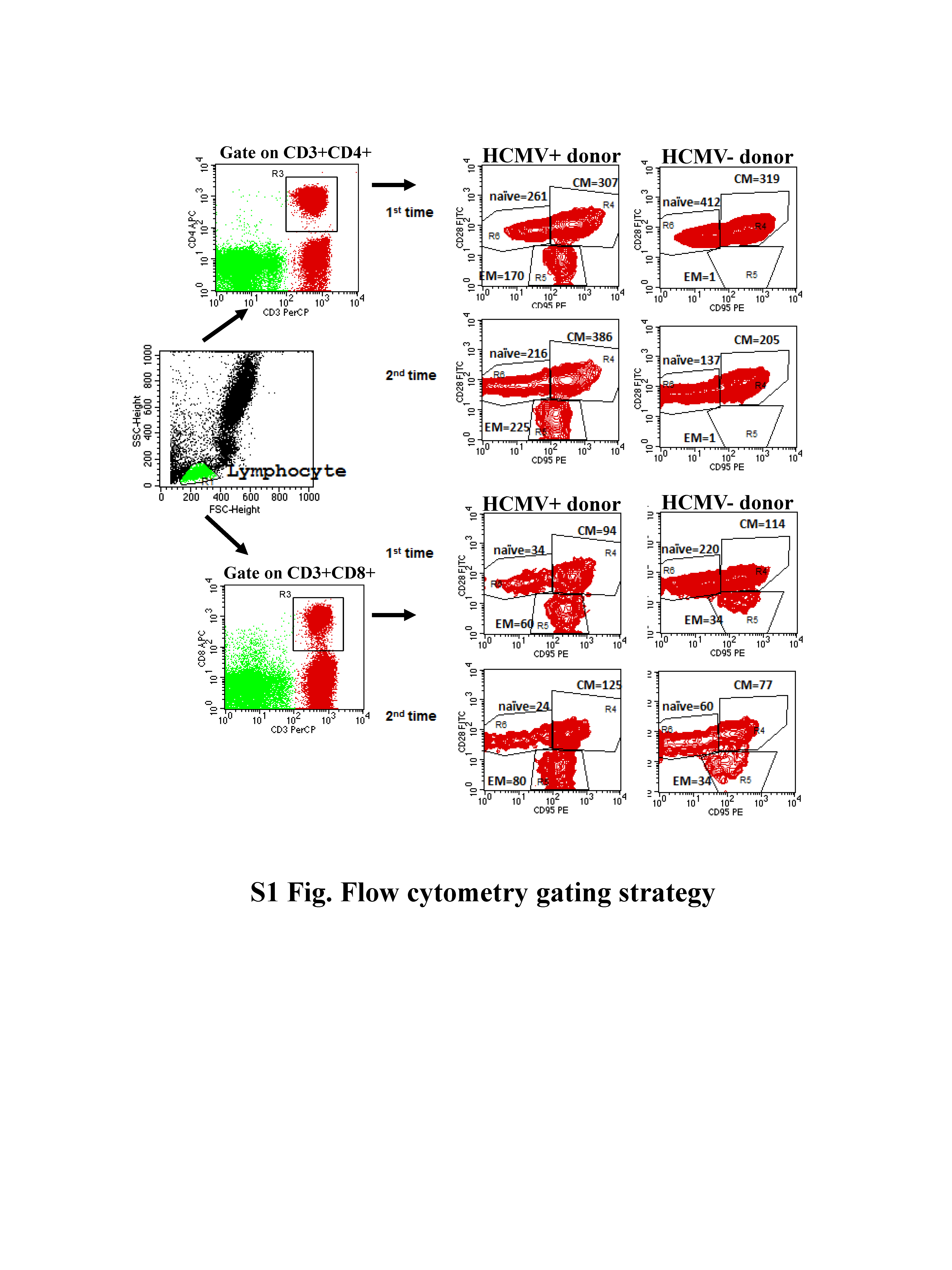

Supplement: S1 Fig — Representative flow cytometric analysis of one HCMV-seropositive and one HCMV-seronegative donor, showing gating to identify T cell subset populations (Naïve, CM, and EM). The absolute numbers of naïve, CM and EM CD4+ and CD8+ T cells per mL of peripheral blood were calculated as described in Materials and Methods. (TIF) [file pone.0151965.s001.tif]

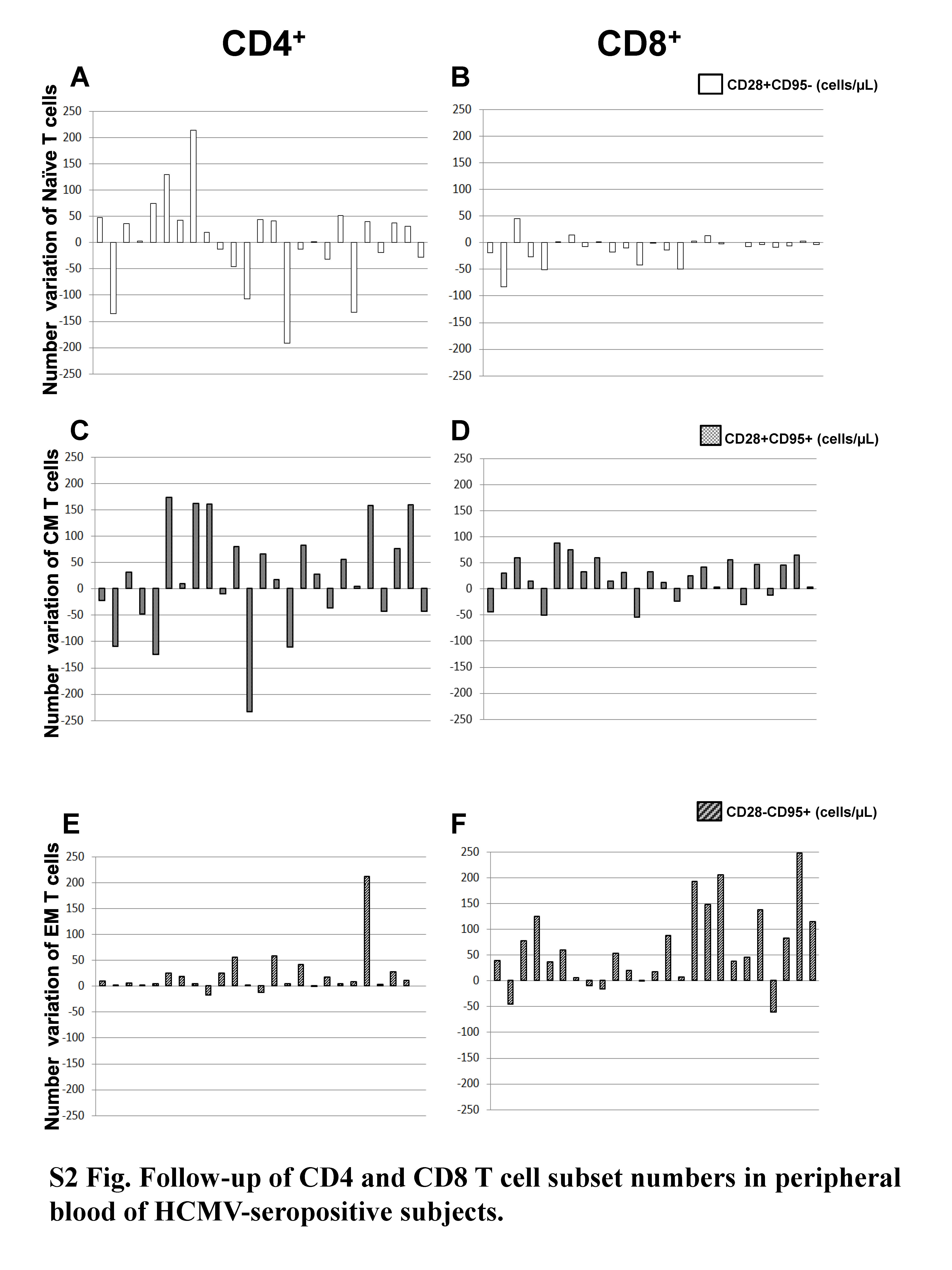

Supplement: S2 Fig — (A, C, E) Number variations of naïve, central memory (CM) and effector memory (EM), among CD4+ T cells and (B, D, F) among CD8+ T cells. The variations are shown in the 25 HCMV-seropositive subjects (from 001 to 025) sorted with ascending order of age. (TIF) [file pone.0151965.s002.tif]

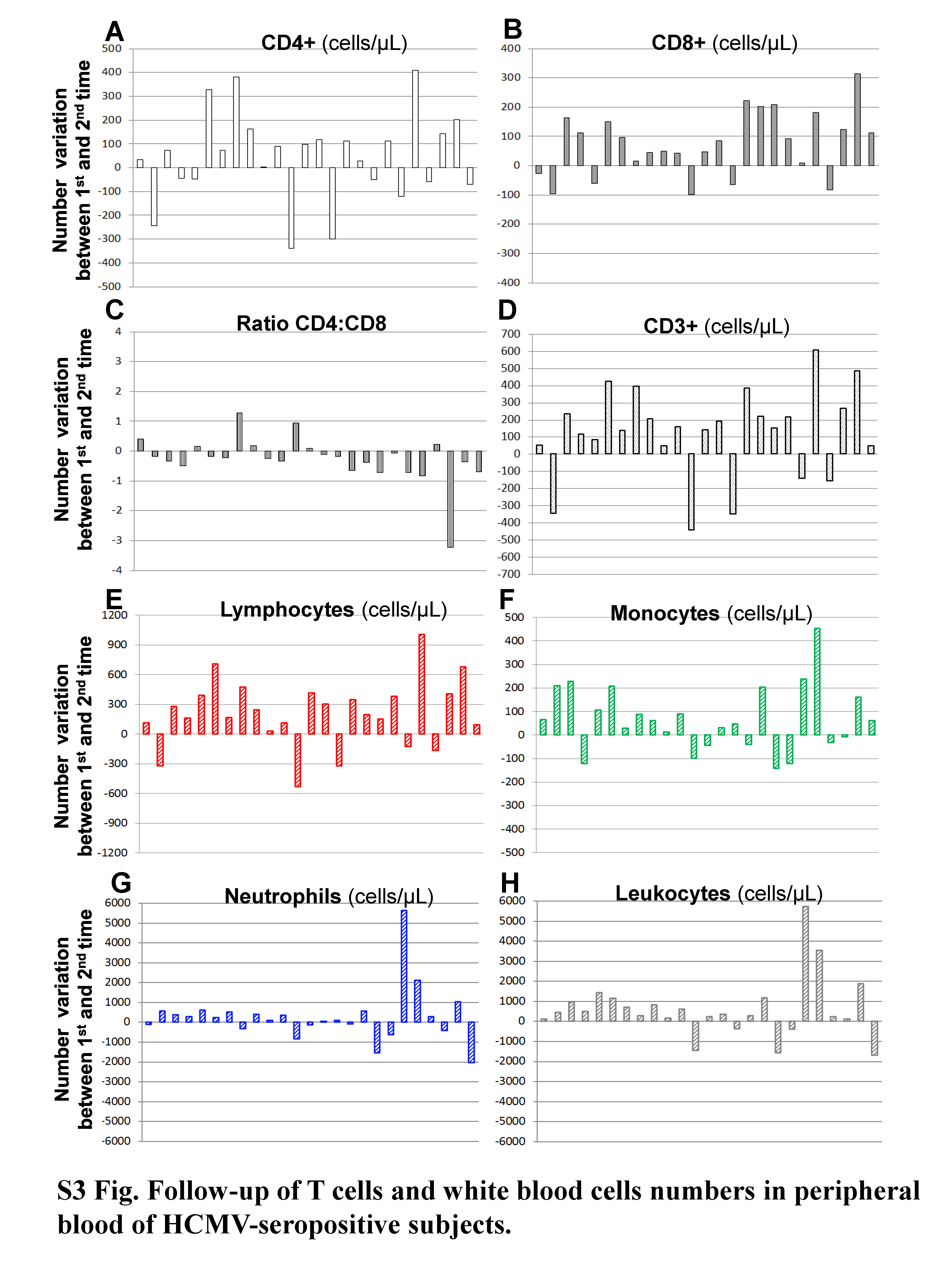

Supplement: S3 Fig — (A, B, C, D) Number variations of total CD4+, total CD8+, ratio CD4:CD8 and total CD3+; (E, F, G, H) number variations of leukocytes, monocytes, neutrophils and leukocytes in the individual 25 HCMV-seropositive subjects sorted with ascending order of age. (TIF) [file pone.0151965.s003.tif]

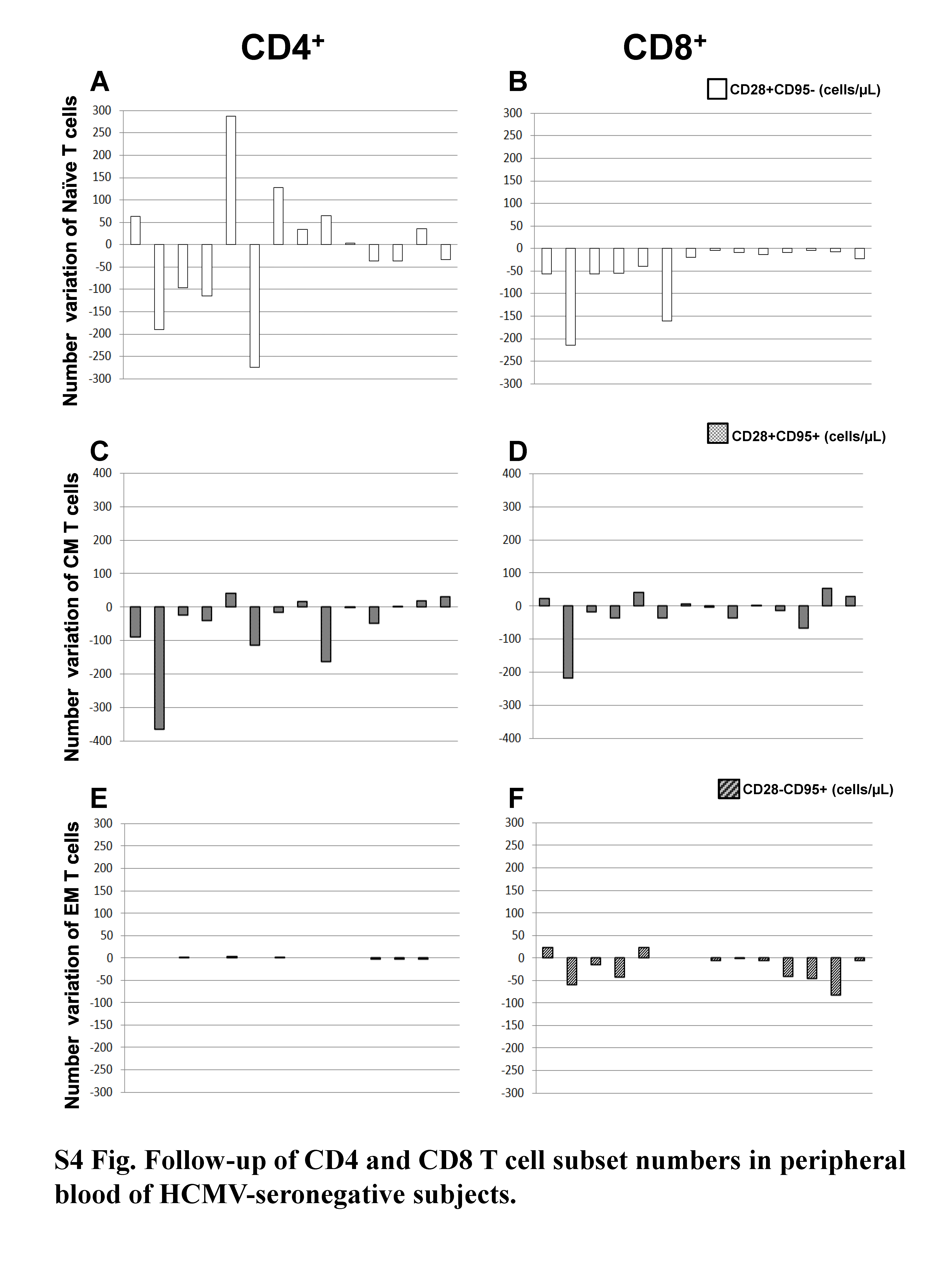

Supplement: S4 Fig — (A, C, E) number variations of naïve, central memory (CM) and effector memory (EM), among CD4+ T cells and (B, D, F) among CD8+ T cells. The variations are shown in the 14 HCMV-seronegative subjects (from 001 to 014) sorted with ascending order of age. (TIF) [file pone.0151965.s004.tif]

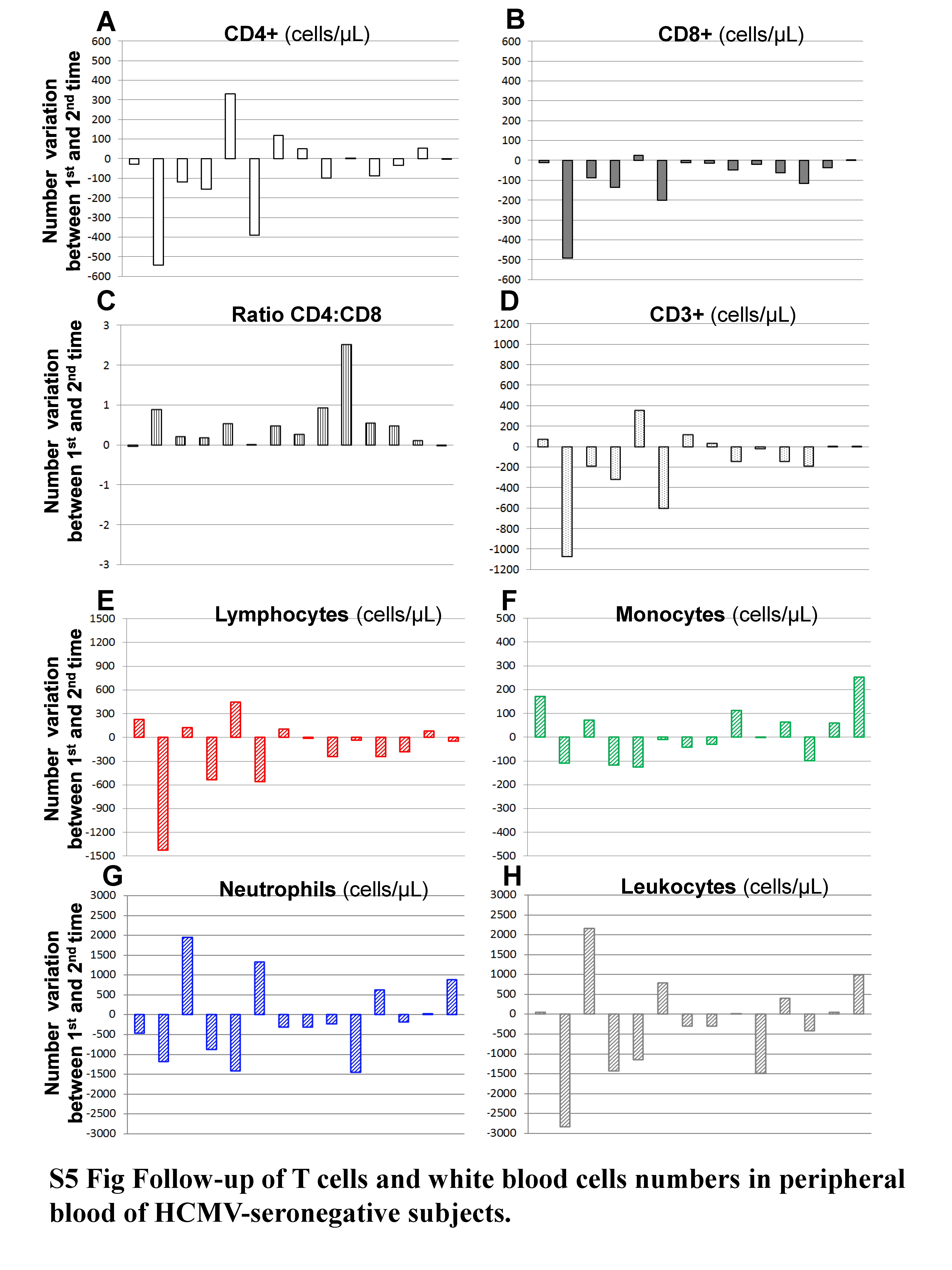

Supplement: S5 Fig — (A, B, C, D) Number variations of total CD4+, total CD8+, ratio CD4:CD8 and total CD3+; (E, F, G, H) number variations of leukocytes, monocytes, neutrophils and leukocytes in the individual 14 HCMV-seronegative subjects sorted with ascending order of age. (TIF) [file pone.0151965.s005.tif]
